# A Probabilistic Model for Cell Population Phenotyping Using HCS Data

**DOI:** 10.1371/journal.pone.0042715

**Published:** 2012-08-23

**Authors:** Edouard Pauwels, Didier Surdez, Gautier Stoll, Aurianne Lescure, Elaine Del Nery, Olivier Delattre, Véronique Stoven

**Affiliations:** 1 Mines ParisTech, Centre for Computational Biology, Fontainebleau Cedex, France; 2 Institut Curie, Paris, France; 3 Institut national de la santé et de la recherche médicale, Paris, France; 4 Institut national de la santé et de la recherche médicale, Unité de Génétique et Biologie des Cancers, Paris, France; 5 Institut Curie, Unité de génétique somatique, Paris, France; 6 The Biophenics Automated Imaging Laboratory, Department of Translational Research, Institut Curie, Paris, France; Chinese Academy of Sciences, China

## Abstract

High Content Screening (HCS) platforms allow screening living cells under a wide range of experimental conditions and give access to a whole panel of cellular responses to a specific treatment. The outcome is a series of cell population images. Within these images, the heterogeneity of cellular response to the same treatment leads to a whole range of observed values for the recorded cellular features. Consequently, it is difficult to compare and interpret experiments. Moreover, the definition of phenotypic classes at a cell population level remains an open question, although this would ease experiments analyses. In the present work, we tackle these two questions. The input of the method is a series of cell population images for which segmentation and cellular phenotype classification has already been performed. We propose a probabilistic model to represent and later compare cell populations. The model is able to fully exploit the HCS-specific information: “dependence structure of population descriptors” and “within-population variability”. The experiments we carried out illustrate how our model accounts for this specific information, as well as the fact that the model benefits from considering them. We underline that these features allow richer HCS data analysis than simpler methods based on single cellular feature values averaged over each well. We validate an HCS data analysis method based on control experiments. It accounts for HCS specificities that were not taken into account by previous methods but have a sound biological meaning. Biological validation of previously unknown outputs of the method constitutes a future line of work.

## Introduction

### Background

**Figure 1:**
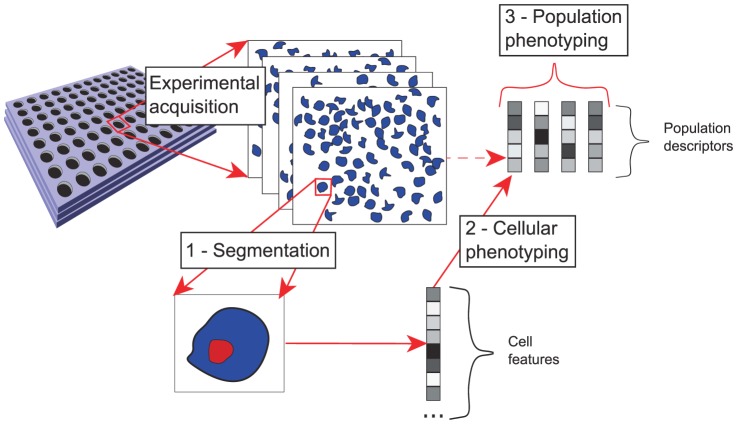
HCS data acquisition and processing. After experimental acquisition, we have four images, or fields, per well. Step 1 consists of isolating each cell in each image and computing cell features by means of image processing tools. These features are used to classify cells in each image according to different predefined cell phenotypes in a second step (for example M, G2 phases or apoptosis). This classification provides population descriptors that can be used to define population phenotypic classes.

Fluorescent markers allow to label virtually any cellular structure in living cells [1]. Recent advances in sample preparation and microscopy automation allow cell population imaging on a large scale [2]. Both technologies lead to the development of High Content Screening (HCS) platforms which allow screening living cells under a wide range of experimental conditions. Classically, the aim is to identify a therapeutic target, or a drug candidate. One screen consists in taking several pictures of a large number of cell populations, for example, transfected with RNAi tools or exposed to small molecules. Each experiment is performed in a well in which several pictures are taken, called fields. It gives access to a whole panel of cellular responses to a specific manipulation. The outcome is a series of cell population images which holds much more information than the single averaged value of the cellular response, classically recorded in HTS screens. The available information accounts for cell variability according to various features, which is precious to characterize a population of cells. However, the heterogeneity of cellular responses makes it difficult to compare and interpret experiments. In the framework we consider in this work, processing the outputs of such experiments requires three steps as illustrated in Figure 1.

- **Step 1, segmentation:** This step consists in identifying cells in images and extract features that characterize the shape and texture for each individual cell.
- **Step 2, cellular phenotyping:** This step usually involves machine learning algorithms that classify cells according to different predefined cellular phenotypes based on cellular features and on a training set of annotated cells for which this phenotype is known.
- **Step 3, population phenotyping:** This step aims at defining phenotypes (or classes) at a population level, using population descriptors derived from cellular phenotypes, in order to describe and compare different experiments.

Segmentation and cellular phenotyping steps have been well studied (steps 1 and 2). There has been a huge amount of work to apply image processing tools to cell segmentation and cell features extraction from cell population images. Typical cellular features used in this context are nucleus and cytoplasm size, texture and shape. The cellular phenotyping step aims at converting, for each single cell, the numerical values corresponding to its cellular features into predefined biological phenotypes that are relevant at the cellular scale and characterize the cell status. Typical examples are cell morphology classes, such as shape and appearance or cell cycle state (G1, S, G2 or M phases). Coupling the image segmentation step with supervised machine learning algorithms, many authors proposed methods to classify cells according to various predefined cellular phenotypes using HCS data and a training set of annotated cells [3]–[5]. These applications developed in the last decade demonstrate empirically the effectiveness of machine learning algorithms in this setting. Example of algorithms used in this context are state of the art classification algorithms such as support vector machines [6] or boosting [7].

A common practice in HCS analysis is to inspect univariate cell features averaged over wells [8]–[10]. This is suited for analysis of a single channel (for example corresponding to a single cellular phenotype such as “apoptosis state”). However, analysis of multiple cellular phenotypes may require to take into account their joint distributions. In our setting, cells from images are phenotyped in steps 1 and 2. As there are many potential cell phenotypes of interest, the multivariate setting must be considered, which constitutes a characteristic of the proposed method. Moreover, our model accounts for the field variability in each well, not only averaged values over wells. This constitutes a step toward cell variability characterization within each well, since within a population of cells in a well, one may observe a range of cellular responses to a given experimental condition. Indeed [11] observe a significant impact of the cell population context on the cellular phenotypes in siRNA screens. They found that local cell density, position of a cell in the local cell population or cell size significantly influence phenotypes such as viral infection or endocytosis.

Once, in an HCS experiment, all cells of a population (namely all cells of a well) have been assigned cellular phenotypes, the aim is to characterize this cell population (step 3). In other words, we would like to define a population phenotype based on the cellular phenotypes of all the individual cells it contains, since different cells taken from a the same well can display different cellular phenotypes, even when exposed to the same experimental conditions. Therefore, cellular phenotypes cannot be used as population phenotypes in a straightforward manner, and it remains a challenging issue to fill the gap between phenotypical characterization of a population of cells and single cell phenotypes. In particular, definition of population phenotypes is an important issue that one must solve in order to compare cell populations subject to different treatments. For example [12] carry out the segmentation and cellular phenotyping steps and propose a distance learning method to compare different cell populations, and generalize known relations between experiments in a third step. In a different experimental setting, [13] use trajectories defined by time varying cell population responses to compare treatments.

### Contribution of this study

In the present study, we develop a method to describe and compare populations of cells in HCS experiments by defining population phenotypes. The input of the proposed method is a table in which each row is a field (an image) and each column is a population descriptor for these fields. For each well several fields are recorded, and well assignment information is available for each field. However, the behaviour of fields within the same well might be different. We refer to this aspect as “within-population variability”. Moreover, population phenotyping should not only take into account each single population descriptor individually, but also the joint distribution of these descriptors. We refer to this as “dependence structure of cell population descriptors”. Taking this dependence structure into account improves the description power of the model. Illustration of these aspects of our HCS dataset and further biological motivations will be presented in the method section.

Going back to the HCS data analysis framework presented in Figure 1, the proposed method tackles step 3: population phenotyping. A natural approach to characterize a population of cells is to consider the output of the first two steps as descriptors for the population of cells. The total number of cells and the proportion of cells assigned to each predefined cellular phenotype describe the joint behaviour of all cells in a given population. The problem is now to assign a population phenotype based on the descriptors of this cells population. For example, in the present study, we aim at defining population phenotypes based on the following population descriptors : cell count, and cellular phenotypes which are represented by proportions of cells in the different stages of the cell cycle. A population phenotype is meant to characterize the biological state of the cell population in a given experiment. Each population phenotype (or class) should gather cells which behaviours are similar, and population of cells showing dissimilar behaviours should be assigned to different classes. The conceptual difference with the cellular phenotyping step (step 2) is that we do not have predefined population phenotypes, nor do we have annotated cell populations according to population phenotypes. Indeed the question of how to define such population phenotypes is still open. Therefore, while a supervised framework is suited for solving step 2, because there exists predefined cellular phenotypes, we propose an unsupervised method to tackle the population phenotyping step (stpe 3) where predefined cell population phenotypes are unknown.

We model a cell population using a hierarchical mixture model which is a specific kind of bayesian network, a widely-used class of probabilistic models [14]. “Within-population variability” is modelled using a hierarchical structure and “dependence structure of cell population descriptors” is modelled using multivariate probability distributions. The output of the method characterizes the density of the input fields in the population descriptors space and assigns a phenotypic class to each field. A copula-based parametrization was compared to a gaussian parametrization of the proposed mixture model (details are found in the methods section). To validate our hypotheses regarding “within-population variability” and “dependence structure of cell population descriptors”, we compare performances of the two preceding models to a baseline gaussian mixture model with diagonal covariance matrix which would correspond to ignoring those two aspects of the data.

In summary, the proposed method is a tool for analysing cell population data. It relies on prior image segmentation and cellular phenotype assignment which corresponds to steps 1 and 2 of this analysis framework. The main purpose of the method is to extract cell population phenotypes and to assess phenotypic variability at the level of cell populations. The model allows to take advantage of HCS specific information: “dependence structure of population descriptors” and “within-population variability”, which our experiments suggest to consider in our context. This can be used to tackle the problem of novelty detection (for example, outlier genes in a siRNA experiment) which is one of the main goals of HCS experiments. We validate a HCS data analysis method based on control experiments. It accounts for HCS specificities that were not taken into account by previous methods but have a sound biological meaning. Biological validation of previously unknown outputs of the method constitutes a future line of work.

## Materials and Methods

### Experimental acquisition

siRNA screening was performed on shA673-1C Ewing sarcoma derived cell line [15] by the Biophenics platform at Institute Curie. Two experimental conditions were considered: cells were either transfected with a negative siRNA controls (Luciferase GL2 siRNA, Qiagen) or a positive siRNA control (KIF11). Cell numeration and mitotic figures were determined using DAPI staining, cycle phases distinction were determined using EdU (for S Phase) and Cyclin B1 (for G2-M transition) immunofluorescence staining. Apoptosis was detected by cleaved caspase 3 immunofluorescence staining. Images were acquired on IN Cell1000 Analyzer (GE Healthcare Life Sciences) and segmented using IN Cell Investigator software.

### Dataset

Our dataset is comprised of 2688 fields belonging to 672 wells for which we have total cell count and proportions in S, G2, M and Apoptotic phases. Each well is either related to a GL2 or a KIF11 experiment. In addition, we have well assignment information for each fields (336 wells×4 wells per fields×2 manipulations = 2688 fields). Note that cellular phenotypes are not exclusive here. This dataset is one example of output of the two first steps we mentioned in the introduction and the purpose of this paper is to validate our method based on it. The proportion of cells in the G0/G1 phases is deduced from the total of those in the S, G2, M.

### Preliminary data analysis

To motivate the need for accounting for “dependence structure of population descriptors” and “within-population variability”, we present two simple observations arising from the dataset described in the previous section.

First we studied the association between cell population descriptors. More precisely, we searched for potential positive or negative correlation between the cell count and the other population descriptors. As shown in Table 1, there is no association between number of cells and S-phase proportion, as expected: DNA replication is a process of quite constant duration because it mainly depends on the species and the size of the genome. Therefore, the length of the S phase should not depend on the proliferation status or the size of the cell population, as observed. There is a slight positive association between cells number and G1/G0-phase proportion. A plausible biological interpretation is that, at a higher number of cells in a well, the population tends to reach confluence, a situation in which the cell cycle is arrested and cells are known to accumulate in phases G0/G1. In addition, we observed stronger dependences between population descriptors. A positive association is observed between cells number and G2-phase proportion, as well as a negative association between cell number and M-phase proportion. This is a biological observation which has not been generally reported, at least to our knowledge. It may be specific to our experimental design, in which the field with the highest number of cells are reaching the limit of confluence and these cells may tend to slow the G2 phase and consequently displaying a reduced number of mitosis. Whatever the interpretation of the above observations might be, these results indicate that the cell descriptors used in this study present a dependent structure, and this justifies the choice of a model that can account for this dependency.

**Table 1:** Association between population descriptors.

	S	G2	M	Apoptosis	G0/G1
rho	0.04	0.51	−0.44	−0.09	0.01
p-value	0.144	2e-16 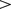	2e-16 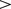	0.0006	0.0004

Association between cell count and proportion of cells in different states based on negative controls. The measure of association is Spearman's rho and the p-value is computed via the asymptotic t approximation [36].

**Figure 2:**
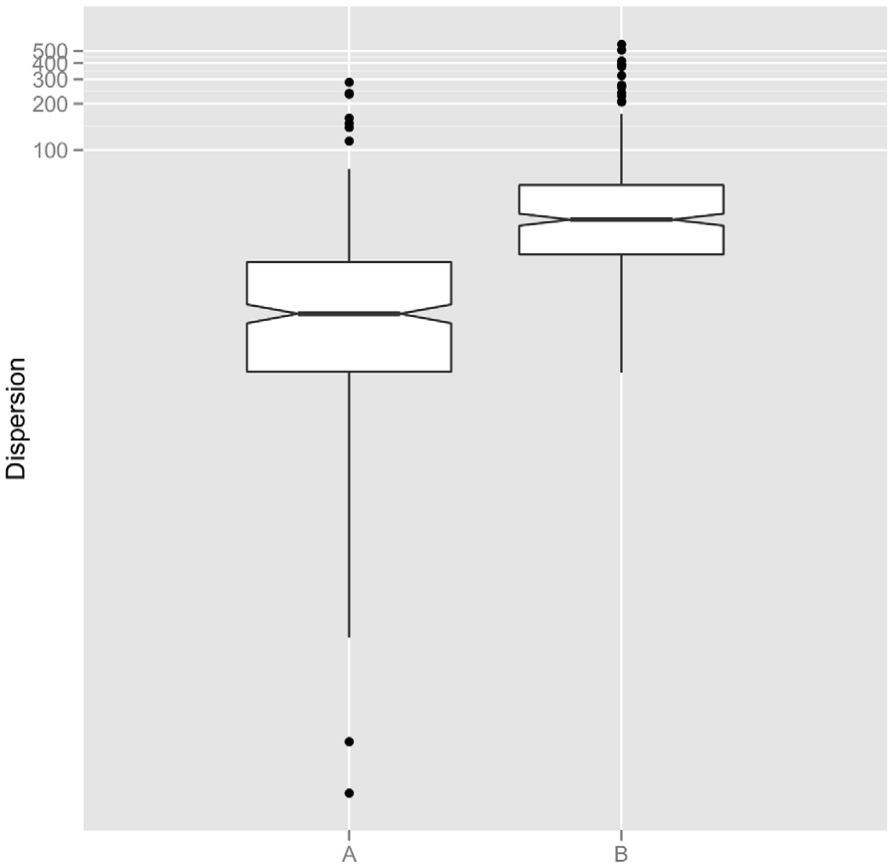
Within population variability. Comparison of the dispersion of fields belonging to the same wells (boxplot A) and randomly selected fields (boxplot B). The measure of dispersion is the sum of squared pairwise distances. The population descriptors (cell count and proportions of cells in S, G2, M and apoptotic states) have been scaled beforehand.

Second, we compared the dispersion of fields belonging to the same well to that of fields randomly selected in the dataset. By dispersion, we mean how close a set of fields are one to the other. The distance used is the euclidean distance and the population descriptors used are cell count and proportion of cells in S, G2, M and apoptotic phases. We scaled the data beforehand and used the measure of dispersion of multivariate analysis of variance proposed in [16]. This is the sum of squared pairwise distances. If we consider the set of fields 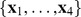, then the dispersion measure is:
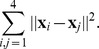
This is equal to the sum of squared distances of each point from the mean, up to a constant multiplicative factor, and therefore measures how dispersed the fields are. Figure 2 indicates that : (i) fields belonging to the same wells do display some variability, which should be taken into account by the model, (ii) this variability is smaller from that of randomly selected fields. Indeed, fields belonging to the same well are part of the same experiment and therefore, are expected to display less phenotypic variability than randomly selected fields. Taken together, these two observations are respectively in good agreement with the ideas of modelling the experimental data taking into account (i) “within-population variability” (ii) within a hierarchical model.

### Model

The proposed model aims at describing HCS data, i.e. a set of wells, each of them containing four fields. The input of the method is a representation based on cell descriptors at the field level (cell count, proportion of cells in S, G2, M and apoptotic phases in our case), coupled to well assignment information. The output is an ensemble of population phenotypes (classes) represented by multivariate distributions. To each image (field), the method assigns a distribution over population phenotypes. We added the quite natural constraint that fields belonging to the same well should correspond to the same class. This hypothesis allows to take into account the “within-population variability” in a given well, which should be part of the population phenotype characterization. This is made possible thanks to the hierarchical structure of the proposed model.

We tested two different parametrizations for this model : copula-based and gaussian-based. Copulas have been studied since the middle of the 20-th century [17] and have been successfully applied to finance [18], hydrology [19], meteorology [20], neurosciences [21] or gene expression data [22]. We introduce copula-based distributions, to build probabilistic densities that represent cell population phenotypic classes. The use of copula for model-based clustering has been suggested by [23], and proposed by [24] in a semi-parametric framework.

#### Formulation of the model

We observe 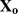 which is composed of 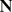 wells 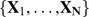. We assume that we have 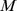 fields in each well 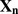. Each field is a vector in 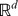. Therefore we represent each well 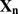 by a 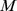-tuple of vectors 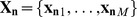 where 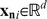 for 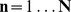 and 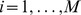. In our application, we have 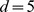 and 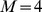. The components of this representation are cell counts and proportions of cells in different phases of the cell cycle. In order to model different classes of wells, we introduce the latent variable 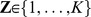 associated to each well where 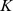 is fixed in advance. We also assume that given the value of 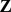, the fields belonging to one well are independent and that wells are independent and identically distributed. These are typical assumptions made in graphical models literature. If 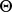 represents the parameters of this model, the density associated to 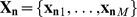 is then
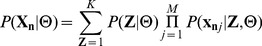
(1)With this definition, the likelihood of the total dataset 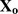 becomes
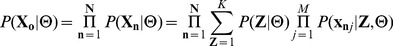
(2)Given 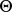, this model can be viewed as a generative process which explains how to generate the data from a probabilistic point of view. To generate a cell population (a well 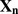), this process takes the following form:

- Choose a population phenotype (a class) from a fixed list. This amounts to sample 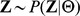.
- Given the population phenotype, generate several sub-populations (fields) according to the multivariate distribution related to this population phenotype. This amounts to sample for 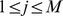, 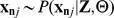

Given 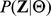 (a multinomial) and the class conditional density 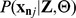, the main issue is to perform inference and learning, which is reversing the generative process defined above to estimate the class distribution related to each well, 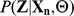, and estimate the parameters of the distributions representing phenotypic classes. We propose gaussian class conditional distributions and copula-based distributions which we now describe.

#### Copula-based class conditional distributions

Copulas became popular in statistical literature at the end of the twentieth century. However, the study of these probabilistic objects goes back to the middle of the century, see [25] for a general review about copulas. The usefulness of copulas comes from Sklar's theorem which states that multivariate distributions can be formalized in term of copula and univariate marginal [26].

We use the gaussian copula family which has been introduced in 2000 by [27]. We use the density function formulation of these copulas which let us work with probabilistic densities. A gaussian copula density function is parametrized by a correlation matrix 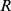. We refer to the gaussian copula density function as 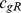. Let 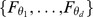 be a set of univariate marginal distributions, 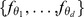 the corresponding univariate densities, such that 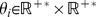 for all 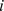. We parametrize 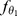 as a gamma distribution with parameters 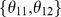. This is a distribution over strictly positive numbers which represents cell counts here. Moreover, we parametrize 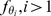 as a beta distribution with parameters 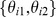. This is a distribution over 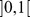 which represents proportions of cells showing different cellular phenotypes. Plugging these marginals in the gaussian copula 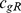, which correlation matrix is 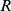, allows to parametrize a distribution which support is exactly the one our variables are limited to, and to model the dependence structure between univariate marginals. If 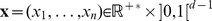, it takes the form:
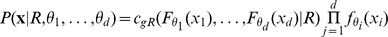
Moreover, we notice that such a parametrization of the class conditional distribution involves exactly the same number of parameters as a standard gaussian model: one correlation matrix and two parameters per univariate marginal. For copula-based densities, standard parameter estimation by maximum likelihood [28] requires computationally intensive numerical optimization. Approximations of this procedure have been proposed to avoid this. Among them, inference function for margin [29], [30] and a semi-parametric procedure [17] which we used to estimate the parameters of our model. This second method consists in using a non parametric estimate of the univariate marginals and computing the copula parameter that maximizes a pseudo-likelihood function. [31] observed empirically that this procedure is more robust to marginals misspecification than the standard maximum likelihood and inference functions for margin. We empirically show in the Results sections that even though this is a crude approximation to maximum likelihood estimator, this parametrization is quite competitive compared to the gaussian one.

#### Inference and learning

Assume that we have a parametrized class probability distribution 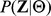 (a multinomial) and a class parametrized conditional distribution 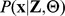, gaussian or copula-based in our case. Finding the best parameters for our mixture model amounts to maximize (2) or the logarithm of (2). Optimizing this objective with respect to 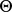 is made difficult by the presence of a sum over latent classes. Approximate inference has shown to be efficient in this kind of setting. [32] provides a general framework for EM type inference among others, which we used to learn the parameters of the model and to infer phenotypic classes of wells in our dataset. Sufficient statistics can be used in the gaussian case. In the copula model case, we implemented the semi-parametric estimation procedure of [17]. After optimizing the model parameter 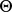, we obtain 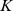 classes represented by class proportions 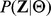 and class distribution 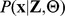. Each well 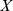 can be represented as a mixture of cell population phenotypes given by 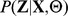, which is inferred during the optimization process.

#### Baseline comparison

The proposed model accounts for “within population variability” through its hierarchical structure and “dependence structure of cell population descriptors” through multivariate probability distributions that model dependence between variables. Those two aspects of the model are motivated by observations arising from the data. In order to validate those hypotheses, we compare the performances of those two models to a standard gaussian mixture model with diagonal correlation matrices. This model does not take into account the fact that different fields come from the same well. It also assumes an absence of dependency between population descriptors, because the gaussian class conditional distribution covariances matrices are constrained to be diagonal.

## Results and Discussion

Data was generated from a siRNA based HCS on a Ewing sarcoma derived cell line. The considered population descriptors were cell count and proportion of cells showing different cellular phenotypes (S, G2, M phase or apoptotic state). From these data, positive and negative siRNA controls were used in this work to illustrate our approach. GL2 siRNA is a negative control that does not affect proliferation and cellular phenotypes. KIF11 siRNA is a positive control that induces cell death and therefore leads to massive alteration of cellular phenotypes.

As presented in the “Preliminary data analysis” of Materials and Methods section, we observed that the population descriptors displayed a dependent structure, and that fields belonging to the same well presented less dispersion than fields randomly selected from the dataset (see Figure 2). These preliminary results justify the use of the proposed gaussian or copula based models.

We first compare the gaussian and copula based parametrizations of the model in terms of model fitting and generalization properties (See model fitting section). Once parameters of the model are fitted to the data, we build an object representing the density of the data we considered. This is useful in term of novelty discovery. In our case, it would correspond to finding cell populations that are different from the negative control population (GL2 silencing siRNA transfected cells), which behaviour is supposed to be hardly affected by this transfection. Confronting a test dataset to the model, evaluating the likelihood of this new data with respect to this model, allows to measure how different from the training set the test set is. We observe that the proposed method allows to separate positive and negative controls (see section “Novelty detection and positive controls”).

Moreover, given the training set, the model classes define the population phenotypes and account for the joint distribution of cell population descriptors. We investigate the properties of these phenotypic classes and underline that the copula based parametrization extracts more meaningful phenotypic classes (see section “Model classes as population phenotypes”). Moreover, we show how those population phenotypes account for different cell behaviours by relating the population phenotypes to cellular phenotypes (see section “Relation between population phenotypes (classes) and cellular phenotypes”).

We discuss the advantage of the proposed model compared to previous approaches focusing on one specificity of the approach, “within-population variability” consideration (see section accounting for “within population variability”). We first describe the cross validation experiment that was carried out to evaluate properties of the model.

### Cross validation

We performed 5-fold cross validation experiments on the negative controls dataset composed of 336 wells which represents 1344 fields. This set is split into five subsets of roughly equal sizes. Each subset is taken in turn as a test set, the model is trained on the remaining four sets, and the likelihood of the test set is then evaluated with respect to the model built with the training set. Because the optimization result relies on the initial parameter value, we performed five random restarts for each fold. This allows to evaluate the generalization performances of the model for the whole dataset. We performed this experiment for the gaussian and copula-based models, as well as the baseline model, for a number of population phenotypes ranging from 2 to 20. The number of classes is a parameter of the proposed method. We repeated this experiment ten times over different splits of the dataset. The model giving the best generalization property, i.e. the model with the highest test likelihood, was then trained on the whole negative controls set and the corresponding classes were analysed.

### Model fitting

The cross validation experiment allows to compare different model performances on this dataset. Because all the proposed model are probabilistic in nature, the first criterion we choose to compare different models is the likelihood computed for a test dataset. We proposed two parametrization of the mixture models, a gaussian and a copula-based parametrization which we review in the method section. We compare those two parametrization to the baseline model using this criterion.

**Figure 3:**
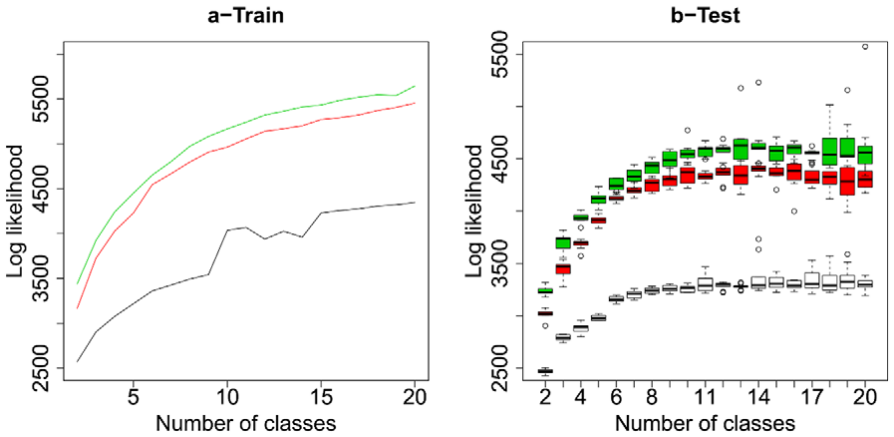
Model fitting. Train (a) and test (b) log likelihood of the negative control data for the two proposed models, and the baseline, varying the number of phenotypic classes. Green corresponds to the copula based model, red corresponds to the gaussian model, and black corresponds to the baseline model. For training log likelihood, we picked the best model among 10 random restarts of the algorithm. For the test log likelihood, the boxes account for the variability among ten different splits of the data in a cross validation setting. Given a data split, for each fold and each number of classes, we picked the best model among 5 random restarts of the algorithm.

Figure 3-a shows the training log likelihood of the two models and the baseline model for different numbers of classes. This training likelihood was evaluated using the whole training set. It appears that the copula-based model results in a higher value of the training likelihood. This observation is valid for the whole range of number of classes we considered. It also appears that the baseline fits much less to training data.

Figure 3-b represents the test log likelihood, evaluated by cross validation, for the two models with different numbers of classes. Again, it appears that the copula-based model has better generalization properties independently from the number of classes. Here again the baseline model provides worse fit on test data.

This experiment shows that the proposed model outperforms the baseline model on both training and test datasets for both gaussian and copula based parametrization. This observation validates assumptions encoded in the model which we referred to as “dependence structure of population descriptors” and “within-population variability”. We consider now comparing in more details the two parametrizations of the proposed model.

The copula-based model outperforms the gaussian model providing better fit on training data and higher generalization properties on a the negative control dataset, while involving exactly the same number of parameters. The copula-based density support matches the domain where our dataset is spread, while the gaussian support is the whole space. Similar results have been reported in other comparative studies of copula models based on different datasets: [33] is an example.

Based on these results, we pick up the model providing the best generalization performances and fit it to the whole negative control set, restarting randomly the algorithm 10 times to avoid local optimum for the parameters values. The results are presented in the two following sections.

### Novelty detection and positive controls

**Figure 4:**
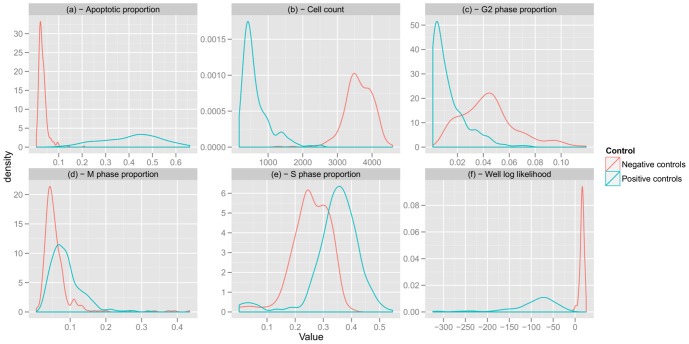
Novelty detection and positive controls. Density plot of cell population descriptors averaged over wells (panel **(a)** to **(e)**) and log likelihood (panel **(f)**) given by the model trained on negative controls. Positive controls are very different from negative controls. It is easy to distinguish them from negative controls only looking at cell count. The log likelihood given by the model separates the two type of controls. We observe that the discriminative power of the univariate descriptors is not lost when considering the model likelihood.

One of the objectives of modelling the negative controls density is to show that we can detect cell populations that are different from these controls, because they could correspond to experiments that are relevant for the studied biological question. To illustrate this point, we used, as controls, cell populations that were transfected with a KIF11 silencing siRNA. We refer to these cell populations as positive controls. It is known that these controls should have a very different behaviour compared to negative controls. Panels **(a)** to **(e)** in Figure 4 represent the densities of positive and negative controls univariate cell population descriptors averaged over wells. Panel **(f)** in Figure 4 represents the densities of positive and negative control log likelihood. Here the model is trained on negative controls.

Positive controls are found to be very different from negative controls. It is easy to distinguish them from negative controls only looking at cell count, for example. The panel **(f)** in Figure 4 represents the distribution of log likelihood over wells. The log likelihood given by the model separates the two types of controls. Training our multivariate model on negative controls and testing it on experiments is not less powerful than using univariate methods.

### Model classes as population phenotypes

We propose to use several densities in a mixture model to define population phenotypes by the classes of the model, which corresponds to a mathematical definition. The number of classes was chosen by cross validation.

**Figure 5:**
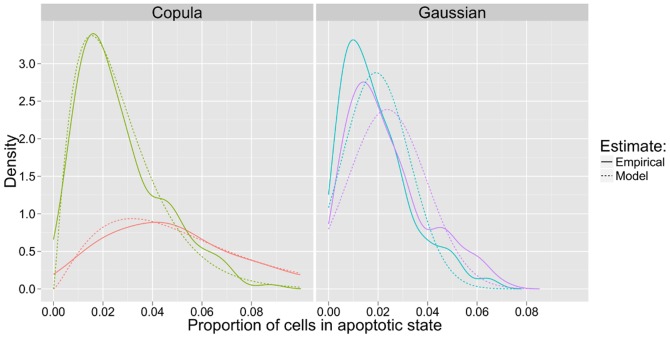
Model and empirical distributions. Examples of classes found by the model (Copula model on the left, gaussian model on the right). The proportion of cells in apoptotic state is represented for the cell populations belonging to those classes. We compare for two classes the univariate marginal densities. For each class the empirical density is represented with a solid line and the density fitted by the model is represented with a broken line.

We inspected the univariate marginal densities. Figure 5 compares the empirical density and the density fitted by the model for one population descriptor, proportions of apoptotic cells, for two phenotypic classes. We notice that the model densities fitted by the copula model are closer to the empirical density compared to those fitted by the gaussian model. In addition, the parameters of the copula distributions represent physically valid distributions. For example, proportions of cells in apoptosis is higher than 0. As shown in Figure 5, the copula-based model accounts for this, while the gaussian model does not.

**Figure 6:**
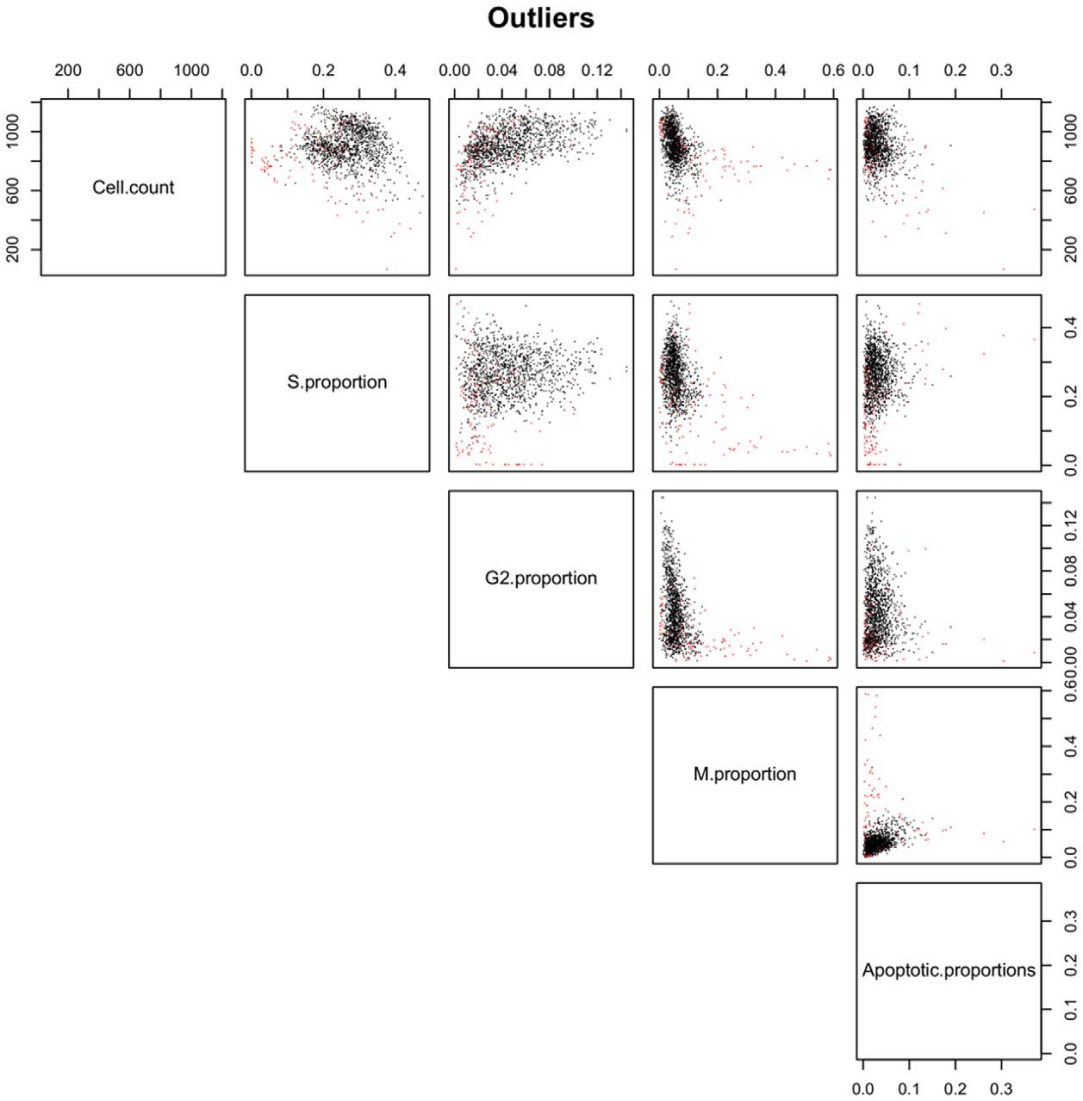
Negative control outliers. Bivariate scatter plots of negative controls. The red points correspond to fields belonging to small classes. They were indeed considered as outliers after checking the images (they were found to be irrelevant). Enough of these wells were present in the dataset so that separate classes were inferred by the model to account for this atypical behaviour.

One example of use of the classes proposed by our model is the detection of atypical behaviours in the training set. Indeed, we inspected visually the cell images of negative control wells that were found in classes containing very few wells (3 classes with 5, 6 and 9 wells respectively over a total of 336). We found that 17 among these 20 wells were not relevant for the negative control modelling because they were experimental outliers. These wells presented a recurrent atypical behaviour, and therefore, a few small classes were inferred to account for this during the learning procedure. Figure 6 shows bivariate scatter plots of the negative control fields and with these outliers. The proposed method provides clues for detection of such cases.

Moreover, we observed that the other classes containing a higher number of wells could account for experimental variability over cell populations. For example some particular classes contained mainly fields in which cell populations had reached confluence, while others did not, as we could observe in the corresponding images. All the classes do not necessarily account for biologically interpretable differences, because the diversity of cell population showing the same behaviours may require several classes to model it accurately. The number of classes was inferred based on cross validation generalization accuracy which is a much more objective criterion.

### Relation between population phenotypes (classes) and cellular phenotypes

**Figure 7:**
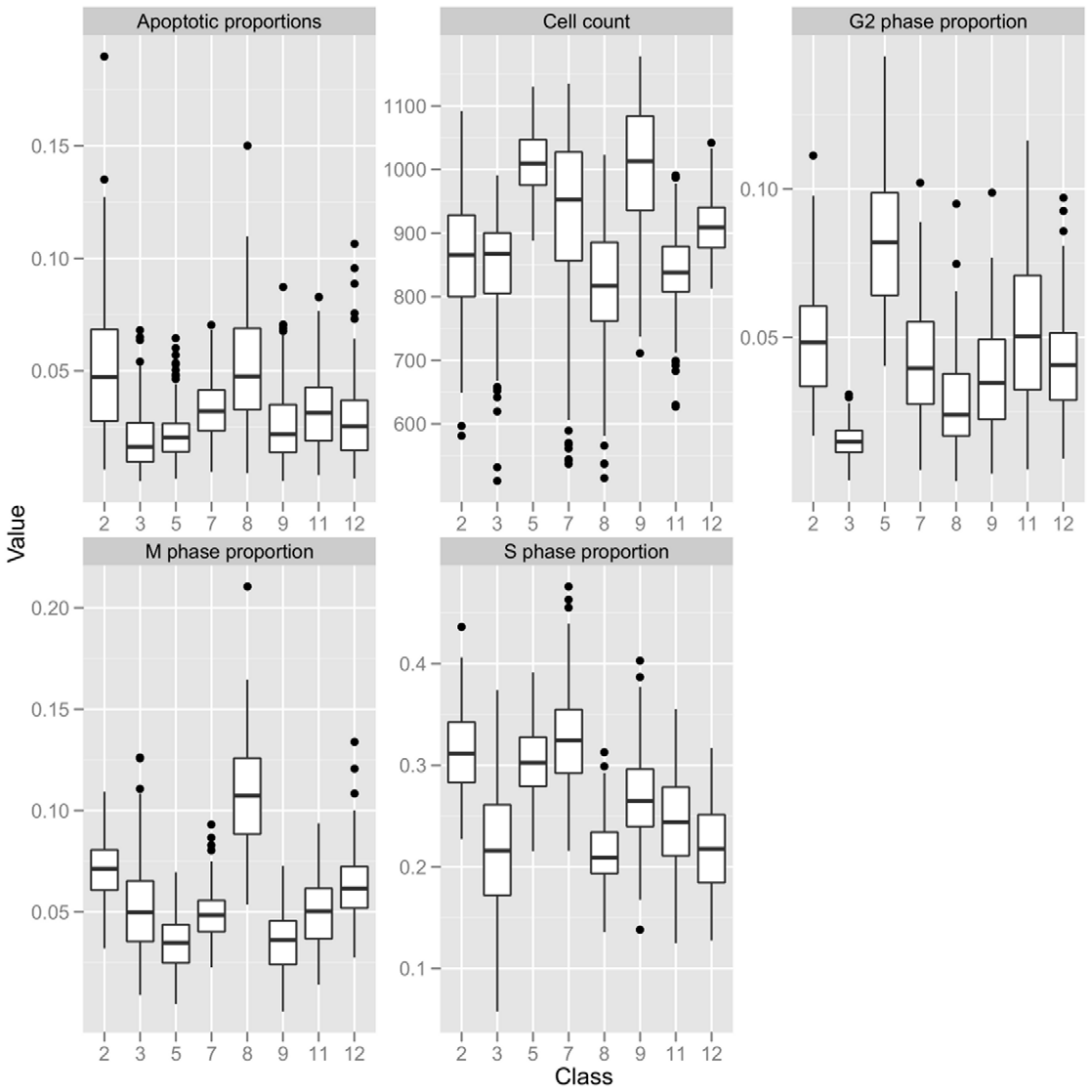
Relation between classes and population descriptors. The classes are represented on the 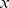 axis. For each class, the boxplot shows the distribution of population descriptors among the fields of this class. Outlier classes were removed. The cell count descriptor has a similar distribution for classes 2 and 3, but other descriptors also allow to differentiate them. Similarly, classes 3 and 5 have have a similar proportion of apoptotic cells, but other descriptors also allow to differentiate them. More generally, each descriptor separates different classes. This suggests that there is no redundancy between population descriptors, and that the classes reflect the combinatorial association between population descriptors.

We considered negative controls and removed the outlier classes since, as mentioned above, they corresponded to irrelevant fields. We inspected differences between remaining classes based on the population descriptors (which were defined from cellular phenotypes), because this could provide some clues about the biological interpretation of population phenotypes. Figure 7 represents the field distribution of population descriptors for each class. It shows that each population descriptor can separates some of the classes, but that none of the descriptors separates all of the classes on its own. This suggests that there is no redundancy between the population descriptors and that the classes reflect possible combinatorial association between population descriptors. The multivariate character of the proposed model allows to account for this fact, while it would not be possible using each population descriptor individually.

### Accounting for “within population variability”

HCS experiments do not provide an average behaviour characterization, but a whole panel of cell responses within different sub populations (fields) taken from the same well. This information is much richer than a simple average response. The data account for the variability of the responses within a given population. As observed in the Materials and Methods section, this variability is not the same as the global field variability. The hierarchical structure of the model allows to take this into account which cross validation suggested to be a correct modeling assumption. Indeed, since all fields of a given well correspond to the same experiment, we therefore impose that they belong to the same phenotypic class. The corresponding density must account for the observed variability between those fields.

**Figure 8:**
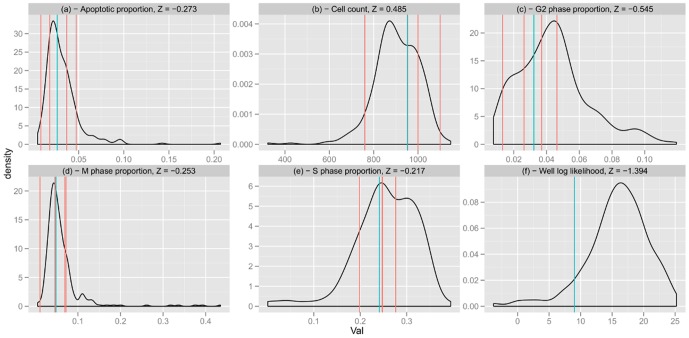
Example of a well. **Panels** (**a**) **to** (**e**), the density plots represent the distribution of cell population descriptors averaged over wells for the negative control dataset. Red lines are the values of the 4 fields of the considered well and the blue lines are the population descriptors averaged over the 4 fields. **Panel** (**f**) represents the density of the log likelihood for all negative controls. The blue vertical line represents the log-likelihood of the considered well.

We illustrate this point in Figure 8 which compares one particular negative control well with the whole set of negative controls. Vertical red bars represented in Figure 8 show that population descriptors averaged over wells do not account for field variability (see Figure legend). Looking at panel **(a)** to **(e)** and blue vertical lines, the well looks similar to the majority of the negative controls. This would correspond to the single descriptor averaged over wells approach. However the red bars in those panels show that there is a lot of variation between the fields taken from this well, and some fields actually fall in tails of the distribution. This is reflected in the **(f)** panel where the vertical blue line is close to the tail of the distribution. Thus the methods could help to eliminate a potential experimental bias while a simpler approach would not.

### Conclusion and future work

In this work, we tackled the cell population phenotyping step in the HCS data analysis framework. This step is performed after image segmentation and cellular phenotyping (steps 1 and 2). It aims at comparing experiments, and gathering cells with similar behaviours in the same class (i.e. assigning them to the population phenotype). The main difficulties in achieving this task are linked to “dependence structure of population descriptors” and “within-population variability” which should be taken into account. Simple observations showed that these are naturally occurring facts observed in our HCS data.

We implemented and compared the performances of two different parametrization of a mixture model, and baseline model that does not account for the specific aspects of the data underlined above. This was performed based on a dataset comprised of two types of cell populations. A comparison of model fitting on test data, using cross validation, suggest that the two specific aspect of the data we focused on when building the model should be considered when studying this kind of data. Moreover, the copula-based parametrization of the proposed model outperforms the gaussian parametrization. However this copula-based model has some disadvantages from the computational point of view, model fitting being much slower and requiring approximations compared to the gaussian formulation.

The main features of cell populations that the model is able to describe are:

- Univariate variables (cell count or cellular phenotype proportions in our case), described by parametric densities
- Multivariate dependence structure, described by a copula
- Variability within a cell population, described by the hierarchical structure of the mixture model

These features constitute the specificity of HCS data. The proposed model takes them into account to build a phenotypic characterization at the population level. Cross validation experiments suggest that taking into account these aspects of the data provides better models. The literature is very scarce regarding population phenotypes definition. To our knowledge, none of the proposed methods take into account the “within-population variability”, which underlines the originality of the proposed model. Pushing this idea further, a future line of work includes modelling at the cell level. [34] propose to infer cell classes from HCS data using single cell measurement. A future work direction is to add a level in the model to infer cell phenotypic classes and population phenotypic classes at the same time in a global model. However the inference computational cost increases a lot and online inference should be used such as in [35].

One application of this model is novelty detection, which is measuring how a cell population related to a given experimental condition is different from a control population. Once a control density is estimated, one can attribute a likelihood to each test experiment which allows to rank them according to how different they are from the controls. For example, the model can detect which siRNA phenotypes are different from a set of controls, and provide orientations toward the most relevant wells in a set of test experiments. The present work constitutes a preliminary validation of this procedure based on two limit cases.

Moreover, the method can help gathering cell populations that show similar behaviours into phenotypic classes. We observed that it can be useful for detection of irrelevant pictures gathered in separate phenotypic classes. The most important future work is to assess to which extend the inferred phenotypic classes are biologically meaningful. For example, wells in which siRNAs target genes with similar biological functions or incubated with drugs with the same target should belong to the same phenotypic class. Future work also include application of the model to target identification. This would require further experimental study for the validation of potential target genes which is far beyond the scope of this paper.

### Reproducible research

Data and source code to reproduce the results presented in this paper are available from http://cbio.ensmp.fr/~epauwels/CellPhen/codeAndData.zip
